# Determinants of institutional delivery in rural Jhang, Pakistan

**DOI:** 10.1186/1475-9276-10-31

**Published:** 2011-07-30

**Authors:** Sohail Agha, Thomas W Carton

**Affiliations:** 1 Population Services International, 1120 19th Street, NW, Suite 600, Washington DC 20036, USA; 2 Department of International Health and Development, Tulane University School of Public Health and Tropical Medicine, 1440 Canal Street, New Orleans, LA 70112, USA

## Abstract

**Background:**

There is expert consensus that delivery at a health facility substantially reduces the risk of maternal death. By increasing the use of antenatal (ANC), postnatal care (PNC) and family planning, the risk of maternal death can be further reduced. There has been little investigation of factors associated with the use of these services in Pakistan.

**Methods:**

A representative household survey was conducted in rural areas of Jhang district, Pakistan, to determine the effect of demographic, economic and program factors on the utilization of maternal health services. Married women who had children ages 12 months or younger were interviewed. Data was collected from 2,018 women on socio-demographic characteristics and the utilization of health services. Logistic regression analysis was conducted to identify the correlates of health services use. Marginal effects quantify the impact of various factors on service utilization.

**Results:**

Parity and education had the largest impact on institutional delivery: women were substantially less likely to deliver at a health facility after their first birth; women with primary or higher education were much more likely to have an institutional delivery. Age, autonomy, household wealth, proximity to a health facility and exposure to mass media were also important drivers of institutional delivery. The use of family planning within a year of delivery was low, with parity, education and husband's approval being the strongest determinants of use.

**Conclusions:**

The findings suggest that rural women are likely to respond to well-designed interventions that remove financial and physical barriers to accessing maternal health services and motivate women by emphasizing the benefits of these services. Interventions should specifically target women who have two or more living children, little formal education and are from the poorest households.

## Background

Recent estimates show that the maternal mortality ratio (MMR) in Pakistan declined from 541 maternal deaths per 100,000 live births in 1990 to 376 maternal deaths per 100,000 live births in 2008 [1]. The pace of this decline is considerably slower than what would be required to meet Pakistan's Millennium Development Goal target of reducing the MMR to one-quarter of its 1990 level, or to 135 maternal deaths per 100,000 live births. Pakistan's MMR remains higher than the average for South Asia. The decline in the South Asian average has been driven by India, which has experienced a rapid increase in skilled birth attendance in recent years. In fact, with the exception of Pakistan and Afghanistan, most other South Asian countries experienced an MMR decline of 50% or greater between 1990 and 2008 [1].

Assessments to determine the influence of programmatic factors on declines in maternal mortality levels or of the effectiveness of strategies to increase skilled birth attendance have been conducted in several South Asian countries [2-6], but not in Pakistan. Indeed, there is a near-absence of studies that have identified the determinants of use of maternal health services in Pakistan.

Based on the available empirical evidence, facility-based delivery at a primary level institution, backed by access to referral-level facilities, is a priority strategy for reducing maternal mortality. The risk of maternal death outside the intrapartum period can be reduced by using antenatal care (ANC), postnatal care (PNC) and family planning [7]. Given that the slow decline in the risk of maternal death in Pakistan, it is important to develop an understanding of factors associated with the utilization of ANC, institutional delivery, PNC and family planning in the period following delivery.

This study identifies the relative impact of factors associated with ANC, institutional delivery, PNC and the use of family planning in the period following a delivery in Jhang district, Pakistan. A performance based financing (PBF) strategy to increase institutional delivery is currently being implemented in Jhang, one of the least developed districts in the Punjab. The present analysis is based on baseline household survey data conducted to monitor the performance of the Jhang PBF intervention.

### Literature Review

Only one prior study has assessed the relative impact of factors associated with the utilization of maternal health services in Pakistan. The study was conducted to assess the effect of mother's education on ANC and the use of modern medical care during delivery. The authors found that, of all variables used in their analysis, maternal education had the strongest impact on utilization of health care [8]. Residence in urban areas at the time of the survey had the second largest impact on utilization of maternal health services. Other variables with significant positive effects included husband's education, a proxy for income [8].

These findings regarding the impact of non-program factors on service utilization are consistent with the literature. Income and wealth are powerful determinants of utilization of health services [9]. Mother's education is thought to influence the utilization of maternal health services through multiple mechanisms: educated mothers may attach a higher value to their health, may be more aware of the benefits of preventive health, may have greater decision-making power, may have greater confidence in dealing with service providers, and may be more willing to travel outside their homes [10,11]. Several studies have found urban residence to be strongly associated with skilled birth attendance but not necessarily with the use of ANC [10,11]. The effect of urban residence may be due to a combination of higher incomes and higher availability of trained providers.

Studies have also highlighted the importance of women's autonomy in influencing health services utilization. Autonomy is defined as the capacity to manipulate one's personal environment through control over resources and information in order to make decisions about oneself or family members [12]. Women's autonomy may extend into areas such as control over finances, decision-making power, and the extent to which they have freedom of movement. A study in a North Indian city found that freedom of movement was an important determinant of using trained attendants at delivery, even after taking into account a range of socio-demographic variables [13]. Although the role of autonomy in determining utilization of maternal health services has not been examined in Pakistan, one Pakistani study found that decision-autonomy was associated with lifetime and current contraceptive use even after controlling for women's education and a range of socio-demographic variables. The study did not find a significant effect of movement autonomy on family planning use [14].

Program-related factors may have an influence on the utilization of maternal health services. Evidence regarding the effectiveness of home visits by outreach workers in increasing the utilization of health services is mixed [5]. A study in Nepal found that health worker home visits were associated with an increase in utilization of ANC and PNC but did not have a significant impact on skilled birth attendance [5]. Several researchers have argued that it is difficult to assess the utility of home visiting programs because they are difficult to quantify [15]. A quasi-experimental study which used outreach workers in India to increase the equity of safer delivery practices found that, while antenatal and newborn practices in the home improved, there was relatively little change in the utilization of health services. Substantial differentials remained in the utilization of skilled birth attendant for delivery, with less than 20% of women in the bottom three quintiles using a trained attendant for delivery. The authors conjectured that financial barriers to the utilization of facility services might have been responsible [6]. However, recent evidence from Tanzania suggests that home visits by community-based workers can increase the utilization of ANC visits and delivery by a skilled birth attendant [16].

In Pakistan, findings from an assessment of the Lady Health Worker (LHW) Program, the government's primary program to improve maternal health through home visits and referrals, indicate that visits by LHWs are associated with an increase in ANC utilization but have no impact on skilled birth attendance [17]. The latter findings are similar to what was observed in Nepal [5].

Access may have an impact on the use of health services when the quality of services offered is high [18]. One study evaluated the impact of increasing health service availability in rural Nepal. During a period of extensive construction of health facilities in rural areas, the number of sub-health posts (facilities staffed by auxiliary health workers) increased 12-fold, from 200 to 2597. Among the services provided at these facilities were maternal health services. About 69% of women in rural Nepal lived within one hour of a health facility The study found that physical access to services had only a limited impact on the use of health care services: simulations showed that if all women lived within one hour of a health facility, the use of prenatal care would increase from 32% to 35% and the use of skilled birth attendants would increase from 10.5% to 11.2%. Possible explanations for the small impact of access on the utilization of maternal health services was low staffing and under-stocking of drugs and equipment at public clinics, and limited functionality of the referral system [4].

Financial barriers posed by high out-of-pocket costs for consultations and transport may be important determinants of utilization of maternal health services, particularly in areas where poverty is high and for services that are more costly. Financing of health service utilization through insurance or other schemes may reduce these barriers [9]. A Turkish study found that the positive impact of health insurance coverage on utilization of ANC and skilled birth attendance was of the same order of magnitude as of maternal education, household wealth and urban residence [10]. Financial barriers to utilization of maternal health services remain substantial in Pakistan: 38% of women who did not have their last birth in a health facility cite the high cost of care as the reason for not doing so [19].

## Methods

### Data

Baseline household survey data was collected from a probability sample conducted in 20 purposively selected union councils in Jhang district where a performance-based financing intervention was to be launched. A union council is the smallest administrative unit in Pakistan. Informed consent was obtained from female respondents prior to interviewing them, consistent with IRB procedures approved by Tulane University Medical Center IRB.

The final sampling unit was women with at least one birth in the 12 months preceding the survey. One hundred households were planned to be randomly selected from each union council, with one mother randomly selected from each household using a Kish grid. To adjust for differences in the populations of the union council (populations range from 17,639 to 27,200), the data was weighted. The data was collected by AcNielsen Pakistan (Pvt.) Ltd. who have been conducting household surveys in Pakistan since 1991.

### Measures

#### Dependent Variables

Four dependent variables were used for this analysis - ANC use, institutional delivery, PNC use, and family planning use. For ANC, women were coded '1' if they made at least three ANC visits and '0' if they made fewer visits. For institutional delivery, women were coded as '1' if they delivered in a public or private health care facility and coded '0' if they delivered at home. For PNC, women were coded '1' if they made a PNC visit and '0' if they did not. Finally, for family planning, women were coded '1' if they were currently using any method of family planning (modern or traditional) and '0' if they were not.

#### Independent Variables

All independent variables included in the analysis are supported by prior literature on the determinants of ANC, institutional delivery, PNC, and family planning use. Variables included in the analysis of ANC, institutional delivery, and PNC were mother's age (categorized as under 20 years, 20-24 years, 25-29 years, and 30+ years), parity (number of living children), mother's education (none, primary, middle, secondary, matriculate or higher), mother's autonomy (low, middle, high), wealth quintile (lowest, second, middle, fourth, highest/fifth), home visit by health worker in the last 12 months, travel time to the nearest health facility (within 15 minutes), mode of travel to the nearest health facility (motorized, on foot, other) and exposure to mass media (exposed to television or radio at least once a week).

Two additional family planning-related variables were included for the analysis of family planning use. These were fertility desires (wanting no more children in the future), and husband's approval of family planning.

Wealth quintiles were created in a manner similar to their creation for the Demographic and Health Surveys. Binary variables were created for household possession of the following items: improved water access, flush toilet, use of natural gas for cooking fuel, radio, television, tape recorder, washing machine, refrigerator, bicycle, motorcycle, telephone land line, cellular phone, DVD player, computer, car, and tractor. Factor analysis was conducted to create a wealth factor score, which was then divided into quintiles.

The variable measuring mother's autonomy was created in a similar fashion. First, mothers were coded '1' if decisions regarding the following were made by her alone or by the couple together and '0' otherwise: small household expenditures (e.g. toothpaste, batteries, etc.); large household expenditures (e.g. television, refrigerator, etc.); expenditures on women's clothes and jewelry; woman's employment outside the home; purchase or sale of property; children's clothes; where to take children in the case of illness; where to take the mother in case of illness; purchase of medicine; children's education; use of contraception; and visits to relatives. Factor analysis was used to create a female autonomy score, which was divided into terciles.

### Statistical Analysis

A multistage process was used to create a base model for the first three dependent variables: the use of ANC, delivery at a health facility and the use of PNC. Bivariate relationships between each independent variable and outcomes were investigated using a binary logistic regression model. Those independent variables found to be significant at the bivariate level were included in a multivariate regression model for each dependent variable. Each independent variable was tested using an improvement chi-square test to determine if the independent variable improved the fit of each model. If an independent variable did not improve the fit of the model, it was dropped. Thus, the most parsimonious model was built for each outcome variable. In order to make the models comparable, however, any variable that remained in the final models for any of the dependent variables was retained in the other models.

The two additional family planning-related independent variables were tested for an association with family planning at the bivariate level. The desire to not have more children, and husband's approval of family planning were associated with family planning use at the bivariate level. These variables were tested for significant improvement in chi-square at the multivariate level in the model for family planning.

## Results

The characteristics of survey respondents are shown in Table 1. Nearly 75% of mothers in the sample were under 30 years of age. About 53% of mothers had three or more children. Nearly two-thirds (64%) of mothers had no formal schooling. About 38% of mothers had received home visits by a health worker during the last 12 months. Travel time to the nearest health facility was 15 minutes or less for half the mothers. However, the usual mode of transport to the nearest health facility for two-thirds (67%) was on foot. Nearly one-quarter of mothers were exposed to the mass media at least once a week. Just over one-third (35%) of mothers had made at least three ANC visits during their last pregnancy. About 41% of mothers had their last delivery in a health facility. Only 17% of mothers made a PNC visit after their last delivery. The use of family planning was very low: only 11% of mothers were using family planning at the time of the survey.

**Table 1:** Characteristics of the sample

	% (n = 2018)
**Maternal Factors**	
Mother's Age	
15-19	9.5
20-24	31.0
25-29	33.9
30 plus	25.7
Parity/Living children	
1	23.5
2	23.7
3	18.8
4	13.7
5	8.7
6	5.1
7 or more	6.5
Mother's Education	
None	64.0
Less than primary	7.1
Primary complete	11.8
Middle	8.3
Matriculate or higher	8.9
**Household Factors**	
Mother's Autonomy	
Low	30.4
Medium	34.2
High	35.3
Wealth Quintiles	
Lowest/First	24.4
Second	15.9
Middle	20.5
Fourth	19.5
Highest/Fifth	19.7
**Program Factors**	
Health worker visited during last 12 months	
No	61.9
Yes	38.1
Travel time to nearest health facility	
More than 15 minutes	49.7
15 minutes or less	50.3
Mode of travel to nearest facility	
Motorized	26.6
Foot	66.6
Other	6.8
Mass Media Exposure	
Less than once a week	75.1
At least one a week	24.9
**Use of Services**	
At least three ANC visits during last pregnancy	
No	65.4
Yes	34.6
Delivered last child at a health facility	
No	58.8
Yes	41.2
Visited health facility for PNC after last delivery	
No	83.1
Yes	16.9
Currently using a method of family planning	
No	88.5
Yes	11.5
Total	100.0

### Factors associated with antenatal care

Table 2 shows factors associated with a woman's making at least three ANC visits during her last pregnancy. Column 1 shows the percentage of women who made at least three ANC visits, by maternal, household and program factors. Overall, just over one-third (35%) of women made at least three ANC visits during their last pregnancy. ANC visits declined markedly with parity: 42% of women with only one child compared to 18% of women with seven or more children made at least three ANC visits. Mother's education was associated dramatic increases in levels of use of ANC: 26% of mothers with no education, 47% with middle level education and 66% with matriculate or higher education made at least three ANC visits during their last pregnancy.

**Table 2:** Determinants of at least three antenatal care (ANC) visits during last pregnancy

	Use of at least three ANC visits during last pregnancy % (1)	Unadjusted odds of at least three ANC visits (2)	Adjusted odds of at least three ANC visits (3)	Marginal effects % (4)
**Maternal Factors**				
Mother's Age				
15-19	36.6	1.00	1.00	
20-24	37.9	1.05	1.09	
25-29	36.9	1.01	1.28	
30 plus	27.0	0.64*	1.06	
Parity/Living children				
1	41.8	1.00	1.00	
2	38.2	0.86	0.89	
3	39.8	0.92	0.95	
4	27.5	0.53***	0.58**	-10.2
5	26.9	0.51**	0.58*	-10.1
6	19.4	0.34***	0.42**	-15.3
7 plus	18.3	0.31***	0.44**	-14.6
Mother's Education				
None	26.0	1.00	1.00	
Less than primary	39.9	1.86**	1.56*	9.2
Primary complete	45.8	2.40***	1.78***	12.2
Middle	47.3	2.56***	1.63**	10.3
Matriculate or higher	65.9	5.48***	2.96***	24.0
**Household Factors**				
Mother's Autonomy				
Low	28.8	1.00	1.00	
Medium	34.8	1.32*	1.25	
High	39.5	1.61***	1.59***	9.5
Wealth Quintiles				
Lowest/First	21.7	1.00	1.00	
Second	29.9	1.54**	1.27	
Middle	32.9	1.78***	1.39*	6.7
Fourth	37.4	2.15***	1.47*	8.0
Highest/Fifth	53.5	4.17***	1.91***	13.9
**Program Factors**				
Health worker visited during last 12 months				
No	31.8	1.00	1.00	
Yes	39.3	1.39**	1.23*	4.2
Travel time to nearest health facility				
More than 15 minutes	30.4	1.00	1.00	
15 minutes or less	38.8	1.45***	1.26*	4.6
Mode of travel to nearest facility				
Motorized	38.2	1.00	1.00	
Foot	34.2	0.84	0.88	
Other	24.6	0.53**	0.70	
Mass Media Exposure				
Less than once a week	30.0	1.00	1.00	
At least one a week	48.7	2.22***	1.41**	7.2
Total	34.6	-	-	-
R-squared	-	-	9.16%	-

Autonomy was associated with a higher proportion of women making at least three ANC visits: 29% of women with low autonomy, 35% of women with medium autonomy, and 39% of women with high autonomy made at least three ANC visits. The percentage of women who made at least three ANC visits increased substantially with the level of household wealth: 22% of women in the lowest wealth quintile, 33% of women in the middle wealth quintile, and 53% of women in the highest wealth quintile made at least three ANC visits during their last pregnancy.

A health worker's visit during the last 12 months was associated with more ANC visits: 39% of women who were visited by a health worker made at least three ANC visits, compared to 32% of women who were not. Women with a shorter travel time to the nearest health facility had a higher level of use of ANC: 39% of women whose travel time to the nearest health facility was 15 minutes or less made at least three ANC visits, compared to 30% of women who lived more than 15 minutes of the nearest facility. Mass media exposure was associated with more ANC visits: 49% of women who were exposed to mass media at least once a week made at least three ANC visits compared to 30% of women who were not.

Column 2 of Table 2 shows the odds ratios and significance levels of relationships at the bivariate level. All relationships between independent variables and the outcome were statistically significant (at p < 0.05).

Column 3 of Table 2 shows the odds of a woman making at least three ANC visits, after adjusting for other variables. After controlling for other variables in the model, parity, mother's education, mother's autonomy, household wealth, visit by a health worker, travel time to the nearest facility, and exposure to mass media had significant associations with a mother making at least three ANC visits.

Column 4 of Table 2 shows marginal effects from a logistic regression estimate of the effect of maternal, household and programmatic factors on making at least three ANC visits during pregnancy. Maternal factors had the highest impact on the likelihood of at least three ANC visits. Compared to women at parity one, the utilization of ANC was 10 percentage points lower among women at parity four, and 15 percentage points lower among women at parity six. The completion of primary education was associated with a 12 percentage point higher use of ANC, while matriculate or higher education was associated with a 24 percentage point increase in the use of ANC.

The impact of household factors on the utilization of ANC was also quite strong. Women with high levels of autonomy had a nine percentage point higher level of utilization of ANC than women with low levels of autonomy. Compared to women from the poorest households, women from households in the middle wealth quintile had a seven percentage point higher level of utilization of ANC and women from households in the highest wealth quintile had a 14 percentage point higher level of utilization of ANC.

Programmatic factors had a significant but somewhat lower impact on the utilization of ANC care. Compared to women from households which had not been visited by a health worker, use of ANC was four percentage points higher among women whose households were visited by a health worker during the last 12 months compared. Living within 15 minutes of a health facility was associated with a five percentage point higher use of ANC. Weekly exposure to mass media was associated with a seven percentage point higher use of ANC.

### Factors associated with institutional delivery

Table 3 shows factors associated with a mother having had her last delivery at a health facility. Column 1 of Table 3 shows the percentage of mothers who had their last delivery at a health facility, by maternal, household and program factors. Overall, 41% of women reported having had their last delivery at a health facility. Institutional delivery varied dramatically by parity, mother's education, household wealth and mass media exposure. About 53% of women delivered their first child at a facility, compared to 28% of women who delivered their sixth child at a health facility. About 33% of women with no education, compared to 48% with who had completed primary education and 73% with matriculate or higher education had their last delivery at a health facility. About 29% of women in the lowest wealth quintile, 39% in the middle wealth quintile and 61% in the highest wealth quintile last delivered at a facility. About 58% of women with weekly exposure to mass media had their last delivery at a facility, compared to 36% of women with less frequent mass media exposure.

**Table 3:** Determinants of use of health facility for last delivery

	Use of health facility for delivery % (1)	Unadjusted odds of facility delivery (2)	Adjusted odds of facility delivery (3)	Marginal effects % (4)
**Maternal Factors**				
Mother's Age				
Less than 19	41.4	1.00	1.00	
20-24	44.4	1.14	1.34	
25-29	40.8	0.99	1.59*	9.9
30 plus	37.8	0.87	2.01**	14.6
Parity				
1	53.4	1.00	1.00	
2	47.0	0.77*	0.72*	-6.7
3	37.8	0.53***	0.46***	-15.6
4	31.8	0.40***	0.36***	-19.8
5	32.6	0.42***	0.37***	-18.8
6	27.9	0.33***	0.31***	-21.7
7 plus	28.2	0.34***	0.32***	-21.4
Mother's Education				
None	33.3	1.00	1.00	
Less than primary	39.9	1.33	1.19	
Primary complete	48.1	1.86***	1.43*	7.7
Middle	59.5	2.93***	2.12***	16.5
Matriculate or higher	72.6	5.25***	2.99***	24.4
**Household Factors**				
Mother's Autonomy				
Low	36.8	1.00	1.00	
Medium	41.0	1.20	1.16	
High	45.2	1.42**	1.39**	7.0
Wealth Quintiles				
Lowest/First	29.2	1.00	1.00	
Second	33.6	1.24	1.04	
Middle	39.4	1.58**	1.28	
Fourth	44.6	1.96***	1.38*	7.0
Highest/Fifth	60.8	3.77***	1.66**	11.2
**Program Factors**				
Health worker visited during last 12 months				
No	38.5	1.00	1.00	
Yes	45.7	1.35**	1.19	
Travel time to nearest health facility				
More than 15 minutes	40.2	1.00	1.00	
15 minutes or less	42.3	1.09	1.02	
Mode of travel to nearest facility				
Motorized	49.4	1.00	1.00	
Foot	38.2	0.63***	0.69**	-8.1
Other	39.1	0.67*	0.70	
Mass Media Exposure				
Less than once a week	35.7	1.00	1.00	
At least one a week	57.7	2.45***	1.65***	11.0
Total	41.2	-	-	-
R-squared	-	-	9.53%	-

Other factors that were associated with institutional delivery were women's autonomy, a health worker's visit during the last 12 months, and mode of travel to the nearest facility. About 45% of women with high autonomy, compared to 37% of women with low autonomy had their last delivery at a health facility. About 46% of women who were visited by a health worker during the last 12 months had their last delivery at a facility, compared to 38% of women who were not visited by a health worker. About 38% of women who travelled to the nearest health facility on foot delivered at a health facility, compared to 49% who travelled to the nearest facility on some form of motorized transport. Column 2 of Table 3 shows that the odds ratios associated with the above relationships were statistically significant at the bivariate level (at p < 0.05).

Column 3 of Table 3 shows the adjusted odds of institutional delivery. After adjusting for other factors, mother's age became a significant predictor of institutional delivery, with women 25 and older being more likely to deliver at a health facility. After adjusting for other factors, a health worker's visit during the last 12 months no longer remained a significant predictor of institutional delivery. The factors that had the most dramatic effects on institutional delivery at the bivariate level - parity, mother's education, household wealth, and mass media exposure - had significant effects on institutional delivery even after controlling for other variables. The effects of autonomy and mode of travel to the nearest facility remained largely unchanged after controlling for other variables, indicating the independent effects of female autonomy and mode of travel on institutional delivery.

Column 4 of Table 3 shows the marginal effects from a logistic regression estimate of the effect of maternal, household and programmatic factors on institutional delivery.

Compared with women under 20 years of age, institutional delivery was nearly 10 percentage points higher among women ages 25-29 and 15 percentage points higher among women 30 and older. Parity and women's education had powerful effects on institutional delivery: compared to women at parity one, delivery at a health facility was seven percentage points lower for women at parity two and 22 percentage points lower for women at parity six. Compared to women with no education, institutional delivery was eight percentage points higher for women who had completed their primary level education, 16 percentage points higher for women with middle education, and 24 percentage points higher for women with matriculate or higher education. High personal autonomy was associated with a seven percentage point increase in institutional delivery. Institutional delivery was seven percentage points higher among women from households in the fourth quintile and 11 percentage points higher among women from households in the highest quintile. Institutional delivery was eight percentage points lower among women whose mode of travel to the nearest health facility was on foot. Institutional delivery was 11 percentage points higher among women exposed to the mass media at least once a week.

### Factors associated with postnatal care

Table 4 shows factors associated with a PNC visit after the last delivery. Column 1 shows the percentage of women who made a postnatal visit after their last delivery. Only 17% of women made a PNC visit after their last delivery.

**Table 4:** Determinants of postnatal care (PNC) visit after last delivery

	Use of PNC visit after last delivery % (1)	Unadjusted odds of PNC visit after last delivery (2)	Adjusted odds of PNC visit after last delivery (3)	Marginal effects % (4)
**Maternal Factors**				
Mother's Age				
Less than 19	17.8	1.00	1.00	
20-24	16.9	0.93	1.08	
25-29	18.6	1.04	1.71*	7.2
30 plus	14.1	0.75	1.78*	8.0
Parity				
1	23.8	1.00	1.00	
2	20.1	0.81	0.76	
3	16.1	0.61**	0.54**	-7.2
4	11.9	0.43***	0.39***	-9.9
5	8.6	0.30***	0.26***	-12.4
6	8.7	0.31**	0.29**	-11.4
7 plus	10.7	0.38**	0.39**	-9.4
Mother's Education				
None	12.2	1.00	1.00	
Less than primary	16.9	1.48	1.23	
Primary complete	20.7	1.88***	1.39	
Middle	28.6	2.89***	1.90**	9.5
Matriculate or higher	34.4	3.78***	1.84**	9.0
**Household Factors**				
Mother's Autonomy				
Low	14.5	1.00	1.00	
Medium	17.2	1.22	1.16	
High	18.5	1.34	1.34	
Wealth Quintiles				
Lowest/First	7.9	1.00	1.00	
Second	15.3	2.13**	1.85**	8.8
Middle	12.6	1.69*	1.39	
Fourth	19.1	2.79***	2.05**	10.3
Highest/Fifth	31.7	5.44***	2.92***	16.4
**Program Factors**				
Health worker visited during last 12 months				
No	16.7	1.00	1.00	
Yes	17.2	1.04	0.91	
Travel time to nearest health facility				
More than 15 minutes	14.4	1.00	1.00	
15 minutes or less	19.4	1.43**	1.13	
Mode of travel to nearest facility				
Motorized	18.7	1.00	1.00	
Foot	16.7	0.87	0.99	
Other	11.6	0.58	0.73	
Mass Media Exposure				
Less than once a week	13.8	1.00	1.00	
At least one a week	26.2	2.22***	1.33*	3.8
Total	16.9	-	-	-
R-squared	-	-	8.75%	-

At the bivariate level, factors that had the largest effect on a PNC visit were parity, mother's education, household wealth and mass media exposure. Women were most likely to make a PNC visit after their first delivery: 24% of women who had one child made a PNC visit after their delivery, compared to 9% of women with six children. Education increased PNC visits: 12% of women with no education, 21% of women who had completed primary level education and 34% of women with matriculate or higher education made a PNC visit after their last delivery. Household wealth was associated with higher PNC visits: 8% of women from households in the lowest wealth quintile, 13% of women with households in the middle wealth quintile and 32% of women with households in the highest wealth quintile made a PNC visit. Shorter travel time to the nearest health facility was associated with higher use of PNC; 19% of women who lived within 15 minutes of their nearest health facility made a PNC visit, compared to 14% of women who lived further away. About 26% of women with weekly mass media exposure made a PNC visit, compared to 14% of women with less than weekly exposure to mass media. The above relationships were statistically significant at the bivariate level (Column 2, Table 4).

Column 3 of Table 4 shows the odds of a PNC visit after controlling for other factors. After adjusting for other factors, age became significantly associated with PNC use, with women older than 25 being more likely to make an ANC visit after their last delivery. Parity, education, household wealth and mass media exposure remained significant predictors of PNC.

Column 4 of Table 4 shows the marginal effects from a logistic regression estimate of the effect of maternal, household and programmatic factors on a PNC visit. Household wealth had the largest impact on making a PNC visit. Compared to women in the lowest income quintile, a PNC visit was nine percentage points higher among women from households in the second income quintile and 16 percentage points higher among women from households in the highest income quintile. Compared to women ages 19 and below, a PNC visit was seven percentage points higher for women aged 25-29 and eight percentage points higher for women 30 and older. PNC visits declined with parity: women with three children had a seven percentage point lower use of PNC while women with five children had a 12 percentage point lower use of PNC. Middle or higher education was associated with a nine percentage point increase in use of PNC. Weekly exposure to mass media was associated with a four percentage point increase in PNC.

### Factors associated with use of family planning

Table 5 shows factors associated with the current use of family planning among women who had a birth during the last 12 months. Column 1 shows the percentage of women who were using a family planning method at the time of the survey. Overall, 11% of women who had delivered during the last 12 months were using a family planning method at the time of the survey.

**Table 5:** Determinants of current use of family planning among women who have delivered during the last 12 months

	Current use of family planning % (1)	Unadjusted odds of current use of family planning (2)	Adjusted odds current use of family planning (3)	Marginal effects % (4)
**Maternal Factors**				
Mother's Age				
Less than 19	7.9	1.00	1.00	
20-24	8.9	1.13	0.83	
25-29	13.2	1.75	0.89	
30 plus	13.3	1.78	0.84	
Parity				
1	4.9	1.00	1.00	
2	10.0	2.17**	1.90*	6.3
3	13.7	3.06***	2.49**	9.4
4	13.4	3.02***	2.33*	8.8
5	16.6	3.85***	3.01**	12.3
6	19.2	4.65***	3.34**	14.1
7 plus	16.0	3.60***	3.38**	14.1
Fertility Desires				
Want more/undecided	7.3	1.00	1.00	
Want no more children	16.3	2.45***	2.16***	6.7
Mother's Education				
None	8.9	1.00	1.00	
Less than primary	10.6	1.24	1.16	
Primary complete	12.6	1.47	1.61	
Middle	18.5	2.34***	2.25**	8.6
Matriculate or higher	22.3	2.98***	2.92***	12.0
**Household Factors**				
Mother's Autonomy				
Low	11.7	1.00	1.00	
Medium	12.7	1.11	0.84	
High	10.0	0.83	0.60**	-4.3
Husband Approval of FP				
No	4.1	1.00	1.00	
Yes	17.0	4.87***	4.42***	11.3
Wealth Quintiles				
Lowest/First	6.5	1.00	1.00	
Second	9.0	1.40	1.38	
Middle	11.4	1.81*	1.63	
Fourth	13.5	2.24**	1.64	
Highest/Fifth	17.6	3.07***	2.06*	7.2
**Program Factors**				
Health worker visited during last 12 months				
No	11.4	1.00	1.00	
Yes	11.5	1.01	0.89	
Travel time to nearest health facility				
More than 15 minutes	7.0	1.00	1.00	
15 minutes or less	15.9	2.50***	2.10***	6.4
Mode of travel to nearest facility				
Motorized	8.0	1.00	1.00	
Foot	12.9	1.72**	1.69**	4.4
Other	10.8	1.37	1.97	
Mass Media Exposure				
Less than once a week	9.8	1.00	1.00	
At least one a week	16.3	1.77***	1.38	
Total	11.5	-	-	-
R-squared	-	-	16.69%	-

At the bivariate level, husband's approval had the most dramatic effect on the use of family planning: 17% of women whose husbands approved of family planning were using contraception, compared to 4% of women whose husbands did not approve. Family planning use increased with parity: 5% of women with one child, 14% of women with three children and 17% of women with five children were using a family planning method. About 16% of women who wanted no more children were using family planning, compared to 7% of who were not or were undecided. Education was associated with higher use of family planning: 9% of women with no education and 22% of women with matriculate or higher education were using family planning. Family planning use increased with household wealth: 6% of women from the poorest households were using family planning, compared to 18% of women from households in the highest income quintile. Shorter travel time to the nearest health facility was associated with higher use of family planning: 16% of women who lived within 15 minutes of a health facility were using family planning, compared to 7% who lived further away. Respondents whose mode of travel to the nearest facility was on foot were more likely to use family planning: 13% of women who traveled to their nearest facility on foot were using a method of family planning, compared to 8% of women whose mode of travel was motorized transportation. Women who were exposed to mass media at least once in a week were more likely to use family planning: 16% of women with weekly exposure to mass media used a family planning method, compared to 10% of women who had less than weekly exposure to mass media. Column 2 of Table 5 shows the odds ratios associated with the above relationships were statistically significant at the bivariate level (at p < 0.05).

Column 3 of Table 5 shows that, after controlling for other factors, parity, desired fertility, mother's education, autonomy, husband's approval of family planning, household wealth, travel time to nearest facility, mode of travel to nearest facility and weekly mass media exposure were significant predictors of family planning use. However, the direction of effects of several variables was the opposite of the direction of effects of these variables on ANC or institutional delivery. For example, women at higher parity were more likely to use family planning. This is consistent with the literature on family planning: as women get close to their desired family size their use of family planning increases. However, we did not expect that women with high autonomy would be less likely to use family planning. Nor did we expect that women whose mode of travel to the nearest facility was by foot, would be more likely to use family planning.

Column 4 of Table 5 shows the marginal effects from a logistic regression estimate of the effect of maternal, household and programmatic factors on the use of family planning. Parity, women's education, and husband's approval of family planning had the strongest effects on family planning use. Compared to women with one child, women with two children had a six percentage point higher use of family planning while women with six children had a 14 percentage point higher use of family planning. Middle level education was associated with a nine percentage point higher use of family planning, while matriculate or higher education was associated with a 12 percentage point higher use of family planning. Husband's approval of family planning was associated with an 11 percentage point increase in family planning use. Women who wanted no more children had seven percentage points higher use of family planning compared to women who wanted no more children or were undecided. Women who lived within a 15 minute walk of the nearest health facility had a six percentage point higher use of family planning compared to women who lived further away. Travel to the nearest health facility on foot was associated with a 4 percentage point higher use of family planning.

## Discussion

This study was conducted to identify the determinants of utilization of maternal health services in a rural district of Pakistan, Jhang, in order to develop strategies to increase the utilization of these services. The determinants of institutional delivery and ANC use were largely similar. Maternal factors, particularly parity and mother's education, had a substantial impact on a woman's making at least three ANC visits during her last pregnancy and delivering at a facility. Being from poor households also made a woman less likely to make three ANC visits and to deliver at a health facility. These findings suggest that interventions which plan to increase institutional delivery should target women with two or more living children, women with low levels of formal education, and women from the poorest households. The substantial effect of belonging to a wealthier household and the effect of greater personal autonomy on institutional delivery suggest that more attention needs to be paid to the role of the husband in supporting the wife's decision to deliver in a health facility.

Women are most likely to have their first birth in a health facility. Interventions should be developed that take advantage of a woman's contact with a health facility at the time of her first birth. Improved interpersonal communications at the health facility at the time of the first birth may be an ideal way of encouraging women to return for later births.

The use of PNC was low compared to the use of ANC and delivery at a facility. Parity, mother's education and household wealth were important determinants of PNC use. Programmatic factors had relatively low impact on a woman's making a PNC visit: exposure to the mass media was the only program variable associated with a PNC visit. Obtaining PNC appears to be a low priority for women in rural Jhang. Efforts to increase PNC use have had relatively little impact in Pakistan.

The use of family planning within a year of delivery was also extremely low: only 11% of women from rural Jhang who had delivered in the last 12 months were using a contraceptive method at the time of the survey. The rate of exclusive breastfeeding in Pakistan is low (only 37% of infants under six months are exclusively breastfed) [19] and breastfeeding is unlikely to provide substantial protection against pregnancy in the first 12 months after delivery.

The low levels of PNC and family planning use are noteworthy. By contrast, women were more likely to use health services for ANC. A woman's contact with health services is most likely to occur during her pregnancy. Interventions should be designed that target pregnant women with information regarding the importance of PNC and family planning use after delivery. Pregnancy is a period in which a woman is likely to be most amenable to being convinced to use services that are beneficial for her and for her child's health.

Parity, a woman's education and husband's approval of family planning were the strongest drivers of family planning use. The lack of association between exposure to mass media and family planning use among recent mothers highlights the importance of making this group a priority for family planning behavior change communications: women who have had recent births have not been targeted by the national family planning program.

## Conclusions

Financial barriers to the utilization of maternal health services and the lack of knowledge concerning the importance of obtaining delivery care from a trained provider remain substantial barriers in Pakistan [19]. Our findings suggest that interventions that lower financial barriers to accessing institutional delivery are likely to have an impact. Barriers related to transportation have an independent effect on lowering the use of ANC and institutional delivery. These barriers should also be addressed as part of health interventions, either by compensating women for transport costs or by providing transportation to reach the health facility.

Efforts should be made to design interventions which increase the use of PNC and the use of family planning after delivery. Interventions that make pregnant women aware of the need for PNC and the use of family planning after delivery are likely to be important, as are interventions that increase husbands' involvement in decision to use these services.

## Competing interests

The authors declare that they have no competing interests.

## Authors' contributions

SA was responsible for the design of the study, the analysis and the write-up of the report. TWC shared responsibility for the analysis and the literature review. All authors read and approved the final manuscript.

## References

[B1] Hogan MC Foreman KJ Naghavi M Ahn SY Wang M Makela S Lopez AD Lozano R Murray C Maternal mortality for 181 countries, 1980-2008: a systematic analysis of progress towards Millennium Development Goal 5 Lancet 2010 10.1016/S0140-6736(10)60518-1 20382417

[B2] McCarthy J Maine M A framework for analyzing the determinants of maternal mortality Studies in Family Planning 1992 23 23 33 10.2307/1966825 1557792

[B3] Ronsmans C Graham WJ Maternal mortality: who, when, where, and why Lancet 2006 10.1016/S0140-6736(06)69380-X 17011946

[B4] Hotchkiss DR Expansion of Rural Health Care and the Use of Maternal Services in Nepal Health & Place 2001 7 39 45 10.1016/S1353-8292(00)00036-8 11165154

[B5] Sharma SK Sawangdee Y Sirirassamee B Access to health: women's status and utilization of maternal health services in Nepal Journal of Biosocial Science 2007 39 671 692 10.1017/S0021932007001952 17359562

[B6] Baqui AH El-Arifeen S Darmstadt GL Ahmed S Williams EK Mannan I Rahman SM Shah R Saha SK Syed U Winch PJ Lefevre A Santosham M Black RE Effects of community-based newborn-care intervention package implemented through two service-delivery strategies in Sylhet district, Bangladesh: a cluster-randomized controlled trial Lancet 2008 371 1936 1944 10.1016/S0140-6736(08)60835-1 18539225

[B7] Campbell OMR Graham WJ Strategies for reducing maternal mortality: getting on with what works Lancet 2006 368 1284 1299 10.1016/S0140-6736(06)69381-1 17027735

[B8] Khan Z Soomro GY Soomro S Mother's Education and Utilization of Health Care Services in Pakistan The Pakistan Development Review 1994 33 1155 1166 12346196

[B9] Wagstaff A Poverty and health sector inequalities Bulletin of the World Health Organization 2002 80 97 105 11953787 PMC2567730

[B10] Celik Y Hotchkiss DR The socio-economic determinants of maternal health care utilization in Turkey Social Science and Medicine 2000 50 1797 1806 10.1016/S0277-9536(99)00418-9 10798333

[B11] Navaneetham K Dharmalingham A Utilization of Maternal Health Care Services in Southern India Social Science & Medicine 2002 55 1849 1869 10.1016/S0277-9536(01)00313-6 12383469

[B12] Dyson T Moore M On kinship structure, female autonomy, and demographic behavior in India Population and Development Review 1983 9 35 60 10.2307/1972894

[B13] Bloom SS Wypij D Das Gupta M Dimensions of women's autonomy and the influence on maternal health care utilization in a north Indian city Demography 2001 38 67 78 10.1353/dem.2001.0001 11227846

[B14] Saleem S Bobak M Women's autonomy, education, and contraceptive use in Pakistan: a national study Reproductive Health 2005 2 1 8 10.1186/1742-4755-2-1 16242030 PMC1277848

[B15] Sweet MA Appelbaum MI Is home visiting an effective strategy? A meta-analytic review of home visiting programs for families with young children Child Development 2004 7 5:1435 1456 10.1111/j.1467-8624.2004.00750.x 15369524

[B16] Mushi D Mpembeni R Jahn A Effectiveness of community based safe motherhood promoters in improving the utilization of obstetric care. The case of Mtwara Rural District in Tanzania BMC Pregnancy and Childbirth 2010 http://www.biomedcentral.com/1471-2393/10/14 10.1186/1471-2393-10-14 PMC2858713 20359341

[B17] Oxford Policy Management Fourth External Evaluation for the National Programme for Family Planning and Primary Health Care 2009 Oxford, UK: Oxford Policy Management

[B18] Acharya LB Cleland J Maternal and child health services in rural Nepal: does access or quality matter more? Health Policy and Planning 2000 15 223 229 10.1093/heapol/15.2.223 10837046

[B19] National Institute of Population Studies (NIPS) [Pakistan] and Macro International Inc Pakistan Demographic and Health Survey 2006-07 2008 Islamabad, Pakistan: National Institute of Population Studies and Macro International Inc

